# Editorial: Human Intestinal Permeability, Mucosal Inflammation and Diet

**DOI:** 10.3389/fnut.2022.894869

**Published:** 2022-04-11

**Authors:** Javier Santos, Giovanni Barbara

**Affiliations:** ^1^Laboratory of Neuro-Immuno-Gastroenterology, Digestive System Research Unit, Vall d'Hebron Institut de Recerca (VHIR), Vall d'Hebron Hospital Universitari, Barcelona, Spain; ^2^Department of Gastroenterology, Vall d'Hebron Hospital Universitari, Barcelona, Spain; ^3^Facultad de Medicina, Universitat Autònoma de Barcelona, Bellaterra, Spain; ^4^Centro de Investigación Biomédica en Red de Enfermedades Hepáticas y Digestivas (CIBERHED), Instituto de Salud Carlos III, Madrid, Spain; ^5^IRCCS Azienda Ospedaliero-Universitaria di Bologna, Bologna, Italy; ^6^Department of Medical and Surgical Sciences, University of Bologna, Bologna, Italy

**Keywords:** intestinal permeability, microbiota, intestinal epithelial barrier, gastrointestinal disorders, methods of evaluation, therapy, nutrition

Intestinal permeability is a physiological function of the gut intended to regulate selectively the traffic of small and large molecules across in order to differentiate self from non self. This ability will determine the establishment of intestinal homeostasis or, in the case of relevant dysfunction, the presumed appearance of local immuno-inflammatory responses. If the dysfunction is perpetuated over time, neuro-immuno-endocrine and microbiota counter-responses shall be generated that could putatively lead to abnormal motor, sensory and secretory responses. There are numerous recent and converging evidences indicating that this cascade of pathophysiological events could be closely involved in the origin of the major symptoms of many digestive diseases, particularly functional bowel disorders, also known today as disorders of gut-brain interaction, but also celiac disease, inflammatory bowel disease, colon cancer, liver cirrhosis and some extra-intestinal disorders such as diabetes mellitus and obesity.

However, the final and incontrovertible demonstration of the participation and order of the sequence of events described is not yet available. Of particular debate is whether barrier dysfunction is a primary event or a bystander phenomenon in the pathophysiology of those diseases. For this reason, intestinal permeability has become one of the hottest topics in modern gastroenterology. The existence of uncertainties has also given rise to an increasingly extensive pseudoscientific and grandiloquent dissemination in the media and social networks of the role of intestinal permeability in numerous pathologies, digestive and systemic, and even to the generation of a fictitious syndrome called the “leaky gut”. Moreover, through this massive propaganda, countless therapeutic approaches and magical solutions to dozens of clinical problems are proposed, which confuse patients and doctors and, for the most part, lack a solid scientific basis.

To help the reader have a better ability to discern the real from the unreal, we have prepared a series of concatenated articles under the Research Topic “Human Intestinal Permeability, Mucosal Inflammation and Diet”.

In the first of these articles, Barbara et al., describes the main mediators and molecular mechanisms governing the passage of luminal solutes and ions across the intestinal epithelial barrier. Authors provide an excellent description of the role of major components of the intestinal epithelial barrier: the mucus layer, the intercellular junctions, the gut immune system and the microbiota and their interplay in regulating the two principal routes of transport, the paracellular and the transcellular pathways to maintain barrier integrity. In the final part of their article, authors review the growing evidence implicating intestinal dysbiosis, immune activation, and barrier dysfunction in several human diseases, including irritable bowel syndrome, inflammatory bowel disease, and gluten-related conditions and analyze briefly the potential beneficial role of some nutritional molecules, including vitamin D, glutamine and short-chain fatty acids to regulate intestinal permeability.

The following article, signed by Vanuytsel et al., focuses on common methods used to assess intestinal permeability in the clinical practice. While there is an open debate on the sensitivity and usefulness of the different existing methods for assessing intestinal permeability, authors of this chapter critically describe technical details, molecules used, as well as pros and cons of *in vivo* and *ex vivo* measurements of both intestinal paracellular and transcellular permeability. In addition, putative blood biomarkers of intestinal permeability, such as intestinal fatty acid-binding protein and zonulin, are also discussed because despite being broadly used, they show limitations that should be accounted for. In the second part of this manuscript, the current evidence of the role of impaired barrier function, based mainly on the above-described methods in the pathophysiology of celiac disease, inflammatory bowel disease, disorders of the gut-liver axis such as chronic alcoholic and metabolic liver disease, and bile acid diarrhea is reviewed.

The next article by Inczefi and Molnár (1) elaborates on the mechanisms leading to dysbiosis and altered intestinal permeability in common gastrointestinal and liver disorders, obesity, chronic respiratory and kidney disease, cardiovascular diseases, neuropsychiatric disorders, bone disease and cancer. Authors pay special attention to the microbial impact on the innate and adaptive immune responses and the role of microbial metabolites in the regulation of intestinal permeability. In connection with this, authors assess the potential role of nutrition in correcting microbial composition and function, and indirectly on intestinal permeability.

The last article by Fortea et al., deals extensively with the therapeutic approach to intestinal permeability disorders. Although there is currently no consensus or clinical guideline to help health professionals in the management of disorders related to intestinal permeability alteration, there is undoubtedly a growing interest from the pharmaceutical industry and other stakeholders and from patients themselves to find evidence-based therapeutic solutions for intestinal barrier dysfunction. In this sense, authors try to shed light on the different therapeutic options, which include, among others, dietary management, nutraceuticals and medical devices such as the mucoprotectant xyloglucan, microbiota and drugs, and epigenetic and exosomes-manipulation, through an objective evaluation of the scientific publications in this field.

## Conclusions and Future Perspectives

The role of permeability alterations in the development of certain intestinal and extra-intestinal diseases and in the origin and severity of clinical manifestations is, although debated, increasingly well known. However, it is necessary to obtain more solid evidence of its critical and primary role, to optimize measurement methods, to discover non-invasive biomarkers, and to obtain new regulatory therapies by conducting controlled clinical trials. Progress in the basic and translational knowledge and management of intestinal permeability will surely enable better options of understanding and confronting this group of common disorders to enhance quality of life of those affected.

## Author Contributions

JS and GB contributed equally to write the article and approved the submitted version. All authors contributed to the article and approved the submitted version.

## Funding

This work was supported in part by Centro de Investigación Biomédica en Red de Enfermedades Hepáticas y Digestivas (CIBEREHD), Instituto de Salud Carlos III, Subdirección General de Investigación Sanitaria, Ministerio de Economía y Competitividad: PI17/0190 (JS), CIBERehd CB06/04/0021 (JS), the DISCOVERIE project, GA no: 848228 (JS, GB). This project has received funding from the European Union's 2020 research and innovation programme under grant agreement No. 848228.

## Conflict of Interest

JS has served as consultant for Noventure SL, Devintecpharma, Reckitt, Ipsen & Pileje and discloses present and past recent scientific collaborations with Salvat, Norgine, Alfa-Sigma, Cosmo, Adare, Ordesa and Danone that do not constitute a conflict of interest in developing the content of the present manuscript. GB has been a consultant, served on the advisory board, or received speaker's bureau fees and/or research support from Cadigroup, Falkpharma, Menarini, Parmalat, Sofar, Zespri, Danone, Yakult, Malesci, Noos, Synergy, Alfasigma, Bromatech, Biocodex, AstraZeneca, Devintec, Formedica, GE Healthcare, Mayoly, Sanofi, Eurekol.

## Publisher's Note

All claims expressed in this article are solely those of the authors and do not necessarily represent those of their affiliated organizations, or those of the publisher, the editors and the reviewers. Any product that may be evaluated in this article, or claim that may be made by its manufacturer, is not guaranteed or endorsed by the publisher.
